# Reconstructive metroplastic myomectomy of an infertile woman

**Published:** 2011

**Authors:** Nader Esmailpoor, Mitra Ahmad Soltani

**Affiliations:** Department of Obstetrics and Gynecology, Alzahra Hospital, Guilan University of Medical Sciences, Rasht, Iran.

**Keywords:** *Lowersegment*, *Metroplasty*, *Myomectomy*, *Reconstructivesurgery*

## Abstract

**Background:** While myoma is the most common pelvic mass of women, most women do not seek screening tests for uterine myoma and if they have any fibroid they are not volunteer for its surgical removal.

**Case**: We present here a novel technique of vascular skeletonization to preserve uterus, making pregnancy possible for an infertile woman with a large uterine myoma, situated in the uterine lower segment.

**Conclusion: **Vascular skeletonization to preserve vessels for a case of myomectomy helped preserve the patient's ability to conceive.

## Introduction

Uterine fibroids (also known as myomas or leiomyomas) are the most common benign solid tumors found in the female genital tract (1). They arise from the muscular part of the uterus. Myomas are divided into three groups: if they migrate towards the abdominal cavity, they are called subserous masses. If they follow the path of the intrauterine cavity, they become submucous fibroids. Masses in the uterine muscle are called intramural. 

Localization of uterine fibroid seems to be an important factor in determining frequency and severity of its symptoms. Submucous fibroids may cause severe clinical symptoms such as excessive bleeding, dysmenorrhoea and a predisposition to reproductive failure. Submucous fibroids are associated with chronic endometritis, and a risk for malignant change and are source of pre-term delivery, abnormal presentation, post-partum haemorrhage and puerperal infections (2). Most submucous fibroids occur at the corporeal sites of the uterine cavity. Some are fundal, others are anteriorly, posteriorly or laterally situated. Small fibroids may also arise from the cornual regions, thus interfering with the utero–tubal junction lumen. A few are located at the cervical canal (3). 

Hysterectomy and laparotomic excision have long been considered the two standard routes of surgical treatment for symptomatic submucous fibroids (4). Myomectomy has been the choice for women who want to preserve their reproductive capacity. However, ten to thirty percent of women who are pregnant despite a large myoma will present with some form of complication during pregnancy such as first trimester losses, pressure symptoms caused by the myoma on the mother and fetus, pain of ‘red degeneration’, premature labor, premature rupture of membranes, malpresentations, retained placenta, postpartum hemorrhage and uterine torsion and higher Caesarean section rate mainly due to obstructed labor and malpresentations (5).

No single technique proven to be superior for treating fibroids (6-8). Laparatomy myomectomy may lead to the development of pelvic post-operative adhesions which may further reduce rather than enhance fertility (9). We present here a case of large uterine myoma, situated in the uterine lower segment of an infertile woman with a novel technique to preserve uterus making pregnancy possible for her.

## ‍Case report

A 32 year old woman of several years of marriage was referred to our gynecology clinic for the chief complaint of infertility with pelvic pain and pressure and frequent episodes of UTI and urinary frequency. Her uterine size was larger than what was expected. An ultrasound examination revealed a solid mass (15 cm by 13.6 cm by 20 cm) situated in the inferior segment of the uterus. 

Hystero salpingography (pic 1-A and B) showed obstruction of the left tube because of the mass. Because of her history of infertility, she was not a candidate for hysterectomy. Myomectomy in this size close to vessels could not be done unless the mass being removed while the vessels preserved. 

**The metroplasty procedure**

A lower segment transverse incision was first performed. The enlarged uterus was lifted through the incision. The fibroid was separated from the wall of the uterus. The uterus was scraped (like the process of sharp debridement) from fibroid tissue so vessels remained at the base. The large myoma was around the inferior segment of the uterus (Pic 2-A). To avoid complete detachment of fundus with cervix after the removal of the mass (pic 2-B), the posterior aspect of the uterine wall was sutured to the posterior aspect of the cervix (pic 2-C and D). A Foley catheter was inserted in the uterus (pic 2-E). Using dissolving suture (Vicryl) in a three-layer closure fashion, lateral walls of the uterus was sutured to the cervix. The anterior wall of the uterus was the last part to be connected to the cervix (pic 2-F).

A HSG of the patient, 6 months later demonstrated that the elongation of the inferior segment of the uterus as well as the pressure effect of the mass on the tube was corrected (pic 3). 

The post-operative recovery was uneventful. No blood transfusion was necessary. She was followed up to her pregnancy. Her pregnancy and elective C/S was uneventful. Her baby is also well. 

**Figure 1:**
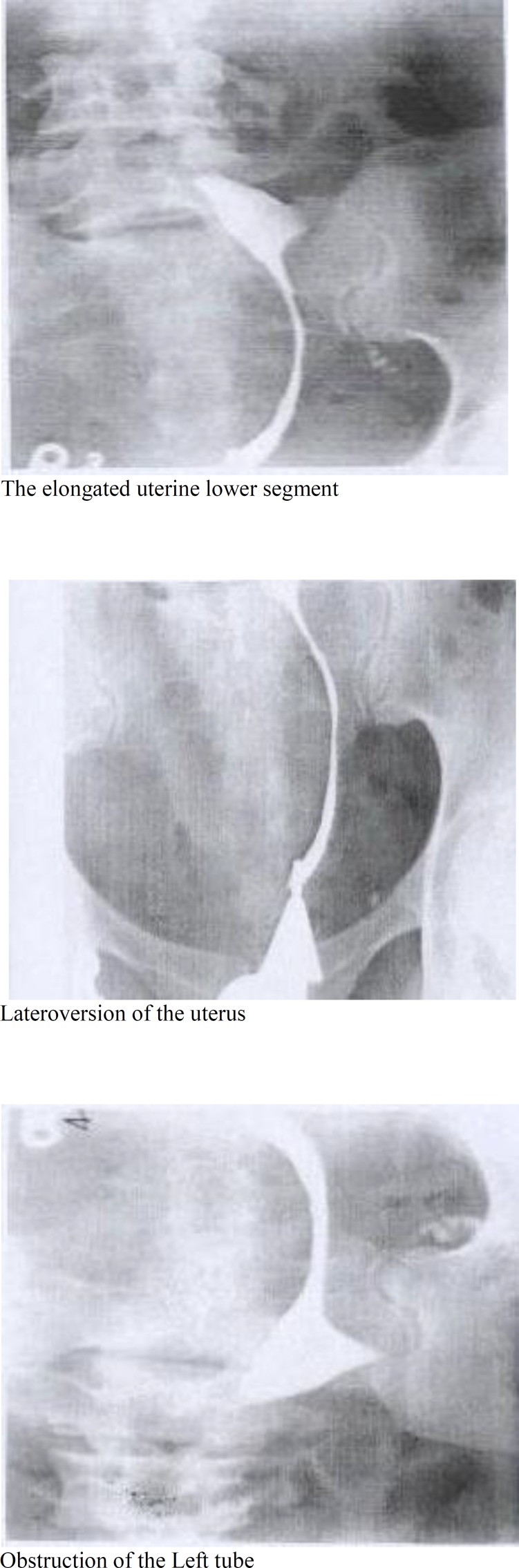
Hystero salpingography of the myoma of the case before the procedure

**Figure 2:**
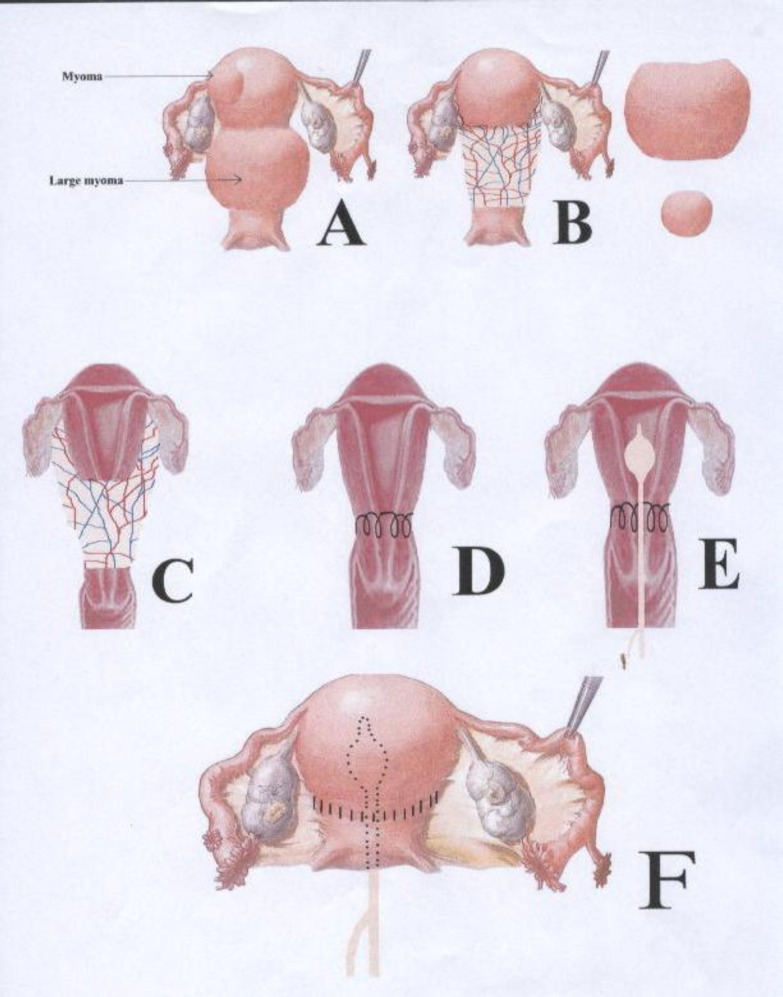
Step by step pictorial description of the procedure

**Figure 3:**
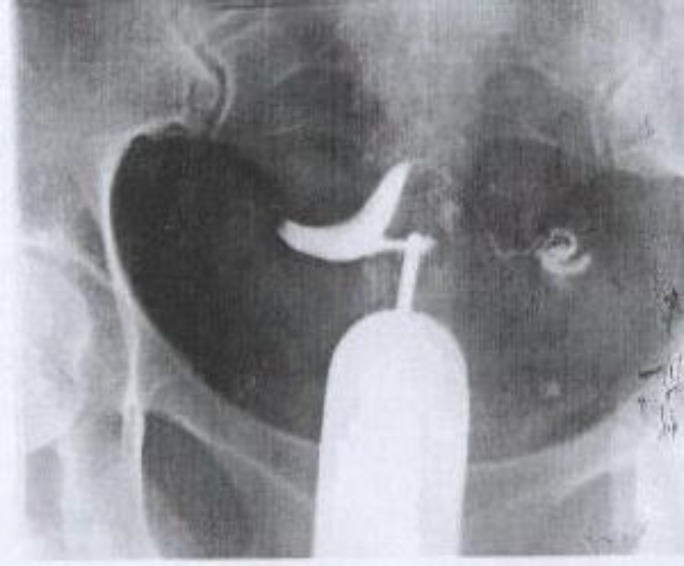
Hystero salpingography of the case after the procedure; tubes are patent and the inferior segment of the uterus is no longer elongated.

## Discussion

Traditionally myomectomy is done by enucleation of the fibroid technically easier in the large fibroids uterus owing to the greater ‘looseness’ of the capsule. A three-layer closure of the incision is advocated because of the large surface area for closure (1). The case described above illustrates that lower segment myomectomy 

(with incisions made over the fibroid) preserving vessels can be safely performed.

Such an operation should only be carried out with optimal anesthetic facilities and by an experienced surgeon who has already had extensive experience in interval myomectomy as the pattern may on occasion be confronted by considerable blood loss.

Sufficient matched blood must be made available along with prophylactic broad spectrum antibiotics. This technique should be considered in carefully selected cases of infertility to allow subsequent pregnancies.
